# GOLPH3 modulates expression and alternative splicing of transcription factors associated with endometrial decidualization in human endometrial stromal cells

**DOI:** 10.7717/peerj.15048

**Published:** 2023-03-20

**Authors:** Suqin Zhu, Dianliang Lin, Zhoujie Ye, Xiaojing Chen, Wenwen Jiang, Huiling Xu, Song Quan, Beihong Zheng

**Affiliations:** 1 Reproductive Medicine Center, Fujian Maternity and Child Health Hospital College of Clinical Medicine for Obstetrics & Gynecology and Pediatrics, Fujian Medical University, Fuzhou, Fujian, China; 2 Medical Research Center, Fujian Maternity and Child Health Hospital College of Clinical Medicine for Obstetrics & Gynecology and Pediatrics, Fujian Medical University, Fuzhou, Fujian, China; 3 Department of Obstetrics and Gynecology, Southern Medical University, Guangzhou, Guangdong, China

**Keywords:** Endometrial decidualization, GOLPH3, RNA-seq, Alternative splicing, Transcription factor

## Abstract

Endometrial decidualization is a decidual tissue formed by the proliferation and re-differentiation of endometrial stroma stimulated by decidualization inducing factors. It is very important for the proper maintenance of pregnancy. Previous studies speculated that Golgi phosphoprotein 3 (GOLPH3) may have a regulatory role in the process of endometrial decidualization, while the specific molecular mechanisms of GOLPH3 is unclear. In this part, GOLPH3 was silenced in human endometrial stromal cells (hESCs), and the transcriptome data (RNA-seq) by GOLPH3 knockdown (siGOLPH3) was obtained by high-throughput sequencing technology so as to analyze the potential targets of GOLPH3 at expression and alternative splicing levels in hESCs. Through bioinformatics analysis, we found that siGOLPH3 can significantly affect the overall transcriptional level of hESCs. A total of 6,025 differentially expressed genes (DEGs) and 4,131 differentially alternative splicing events (DASEs) were identified. Through functional cluster analysis of these DEGs and genes where differential alternative splicing events are located, it is found that they are enriched in the PI3K/Akt signaling pathway, RNA splicing and processing, transcription factors and other pathways related to endometrial decidualization and important biological processes, indicating the important biological function of GOLPH3. At the same time, we focused on the analysis of the transcription factors regulated by GOLPH3, including gene expression regulation and the regulation of variable splicing. We found that GOLPH3can regulate the expression of transcription factors such as LD1, FOSL2, GATA2, CSDC2 and CREB3L1. At the same time, it affects the variable splicing mode of FOXM1 and TCF3. The function of these transcription factors is directly related to decidualization of endometrium. Therefore, we infer that GOLPH3 may participate in endometrial de membrane by regulating expression and alternative splicing levels of transcription factors. We further identified the role of GOLPH3 in the transcriptional mechanism. At the same time, it also expands the function mode of GOLPH3 protein molecule, and provides a theoretical basis for downstream targeted drug research and development and clinical application.

## Introduction

Maternal endometrial decidualization plays an essential role in the establishment of successful pregnancy. Decidualization is the transformation of morphology and function of endometrial stromal cells (ESCs) in order to allow embryo implantation during the window of implantation (Gellersen, Brosens & Brosens, 2007). Decidualized endometrium plays an important role in avoiding maternal immune rejection and the healthy continuation of pregnancy. The understanding and research of endometrial decidualization is of great significance to improve endometrial receptivity (Okada, Tsuzuki & Murata, 2018), and avoid reproductive failure or endometriosis (Gellersen & Brosens, 2014). Endometrial decidualization is regulated by a complex and multifactorial network. The most well studied proteins are progesterone and its nuclear receptor (PGR) that drives the development of endometrial receptivity. This process is triggered by the interaction between progesterone and LBD of progesterone receptor at the target cell plasma membrane, resulting in progesterone receptor translocating into nucleus and regulating transcription of downstream target genes with the participation of coregulators (DeMayo & Lydon, 2020). Cyclic adenosine monophosphate (cAMP), as decidual markers, was increased during decidualization and was correlated with PRL and IGFBP-1 expression (Gellersen & Brosens, 2003). Other coregulators, including homeobox genes (Ashary, Laheri & Modi, 2020), FOXO1 (Kajihara, Brosens & Ishihara, 2013), and HAND2 (Huyen & Bany, 2011), also participate in the process of endometrial decidualization as transcription factors. Meanwhile, further studies are needed to investigate the potential regulators during decidualization to deeply understand the underlying mechanisms.

Recent studies using high throughput sequencing technology have identified genetic mutations and differentially expressed genes associated with recurrent miscarriage. Two well identified genes are PDZD2 and GOLPH3with copy number variants (Nagirnaja et al., 2014). GOLPH3, a Golgi phosphoprotein, is enriched in the Golgi stack and has regulatory functions in Golgi trafficking. GOLPH3 is famous as an oncogene by playing various roles in the progression of multiple cancers (Sechi et al., 2020). The genome location of GOLPH3 (chr5p13) is frequently amplificated in many cancers (El-Maqsoud et al., 2015); GOLPH3 protein modulates cell size, promotes growth-factor-induced mTOR signaling in human cancer cells, and changes the response to an mTOR inhibitor rapamycin *in vivo* (Scott et al., 2009). GOLPH3 has the ability to promote tumorigenicity of breast cancer cells and it has an important relationship with the prognosis of breast cancer patients, perhaps by suppressing expression of FOXO1 (Zeng et al., 2012). Another important function of GOLPH3 is its response to DNA damage, enhancing cell survival and proliferation, and finally promoting development of cancers (Buschman, Rahajeng & Field, 2015). All these results indicate that GOLPH3 is an oncogene localizing on Golgi by regulating the functions of other interacting proteins (Rizzo et al., 2017).

However, besides the copy number variants of GOLPH3 observed in idiopathic recurrent miscarriage (Nagirnaja et al., 2014), very few studies reported the association between GOLPH3 and endometrial decidualization. One recent study demonstrated GOLPH3 mRNA level was dysregulated in Pgr epithelial deleted adult mice uterus compared with control, which may be related to pathways such as cell adhesion, extracellular matrix, and cell proliferation (Gebril et al., 2020). Another study also showed Golph3 mRNA level was higher expressed before blastocyst implantation compared with that after blastocyst implantation in mouse uterine luminal epithelium cells, suggesting the potential regulatory roles of GOLPH3 during endometrial decidualization (Aikawa et al., 2022). Endometrial decidualization is a biological process coupled with cell proliferation, extracellular matrix organization, and cell invasion (Gellersen & Brosens, 2014), which are also observed in cancer development and associated with the functions of GOLPH3 in cancers. We propose GOLPH3 may also play important functions during the process of endometrial decidualization.

In this study, we extensively investigated that the expression level of GOLPH3 in the uterus of model mice and its association to embryo implantation, decidualization and estrogen and progesterone regulation. We also performed experiments to check the influence on proliferation, differentiation, apoptosis, decidualization, and embryo implantation of human endometrial stromal cells (hESCs) by silencing expression level of GOLPH3. In order to explore underlying molecular mechanisms, the whole transcriptome data affected by GOLPH3 was obtained by high-throughput sequencing technology (RNA-seq), and then the potential targets and signal pathways of GOLPH3-regulated genes at transcriptional and alternative splicing levels were analyzed.

## Materials and Methods

### Animals and methods

Sexually mature female Kunming mice weighing 25–30 g were purchased from Fujian Medical University Experimental Animal Center. According to the ratio of 1:1, the mature female and male rats were mated before 18:00 on the same day. The female rats were checked for vaginal plugs at 08:00. Dissect mice at 8:00 to collect mouse uterine tissue for 1–8 days gestation. Immunohistochemistry detected the expression of GOLPH3 protein in uterine tissues of pregnant mice. All animal experiments were approved by the Ethics Committee of Fujian Maternity and Child Health Hospital (No. 2023KYLLRD01079).

### Cell culture and *in vitro* induction of decidualization

hESCs was maintained in our laboratory. The cells were cultured in DMEM medium containing 10% FBS in a 5% CO2 cell incubator at 37 °C. hESCs were treated with 2.0% complete medium containing 0.5 mM8-Br-cAMPand luM MPA for 4 days, and the medium was changed every 2 days. PRL and IGFBP-1, markers of decidualization in human endometrial stromal cells, were detected by Western blot and real-time fluorescence quantitative PCR (RT-qPCR) to verify the success of inducing decidualization.

### Small interfering RNA (siRNA) transfection

ThesiRNA for the GOLPH3 were designed and synthesized by Hippo Biotech (Zhejiang, China). 1, GOLPH3siRNA sequence: sense 5′-GCAGCGCCUCAUCAAGAAAGUdTdT-3′, anti-sense 5′-ACUUUCUUGAUGAGGCGCUGCdTdT-3′. A total of 2, siRNA NC sequence: sense 5′-UUCUCCGAACGUGUCACGUTT-3′, anti-sense 5′-ACGUGACACGGAGAATT-3′. A total of 150 μLOpti-MEM (Thermo Fisher, Waltham, MA, USA) was mixed with 3 μL siGOLPH3 or 3 μL control siRNA (NC), and another 150 μL Opti-MEM (Thermo Fisher, Waltham, MA, USA) was mixed with 9 μL transfection reagents. The above two suspensions were mixed with 150 μL each and left for 5 min. A total of 2 mL complete medium was added into each well of the 6-well plate, and 250 μL of the above suspension was added. RNA and protein were collected after culture for 24–48 h, or were replaced with deciduating induction solution for 2 or 4 d induction on the second day after transfection, for subsequent experiments.

### RNA extraction and sequencing

RQ1 DNase (Promega, Madison, WI, USA) was used to remove DNA and extract total RNA. We then analyzed the purified RNA using BioRad’s Smartspec Plus (A260/A280; BioRad, Hercules, CA, USA) to measure its quality and quantity. We used integrated RNA for following experiment by 1.5% agarose gel electrophoresis method. RNA was captured using VAHTS mRNA capture beads (N401; Vazyme, Nanjing, Jiangsu, China). The purified RNAs were then used to prepare RNA-seq libraries using KAPA Stranded Kits for Illumina^®^ platforms. We processed the cDNA products at −80 °C after size selection (300–500 bps). The cDNA libraries were then used for high-throughput sequencing (RNA-seq) using Illumina Novaseq 6000 system, and finally 150 nt paired-end sequencing reads were obtained.

### RNA-seq data processing and analysis

We sequenced GOLPH3 knockdown human endometrial stromal cells and transfected empty vector-transfected human endometrial stromal cells. Each group was repeated three times, and at least 10G of each sample was measure. According to RNASeqPower, this experimental design has a statistical power of 0.75. Adaptor trimming and quality control of the raw sequencing data were carried out using fastp (0.23.2) (Chen et al., 2018). Fastp (0.23.2) was used to trim and quality check the raw sequencing data (Kim, Langmead & Salzberg, 2015), then transcripts abundances were quantified by featureCounts (2.0.3) (Liao, Smyth & Shi, 2014). Differentially expressed genes were calculated using the DESeq2 package (1.34.0) (Love, Huber & Anders, 2014) with screening thresholds of |log2foldchange| ≥1 and *P*-value <0.05. For alternative splicing events (ASEs) analysis, we used ABLas pipeline. ABLas detected ten types of ASEs using the reads from splice junctions, including exon skipping (ES), alternative 5′ splice site (A5SS), alternative 3′ splice site (A3SS), mutually exclusive exons (MXE), mutually exclusive 5′UTRs (5pMXE), mutually exclusive 3′UTRs (3pMXE), cassette exon, A3SS&ES and A5SS&ES. Student’s t-test was used to evaluate the significance of the ratio alteration of regulated ASEs (RASEs) between samples. A RASE was defined as an ASE with a *P*-value cutoff equal to a false discovery rate cutoff of 5% and ratio change greater than 0.12. By clusterProfiler package (4.2.2), Gene Ontology (GO) and Kyoto Encyclopedia of Genes and Genomes (KEGG) enrichment analyses are performed for differentially expressed genes (Yu, 2018), and Fisher’s exact test was used to estimate the statistical significance of pathway enrichment.

### Statistical method

The data were analyzed by GraphPad prism application software, and the data were presented by mean ± standard deviation. The statistical difference between groups was evaluated by two independent sample t-test or multiple comparison single factor difference analysis. *P* < 0.05 by Student’s t-test indicates the difference is significant.

### Data availability

The RNA-seq data discussed in this article have been published in NCBI’s Gene Expression Omnibus (GEO series accession number GSE215155).

## Results

### Changes in transcriptional levels caused by knockdown of GOLPH3 in hESCs

**Figure 1:**
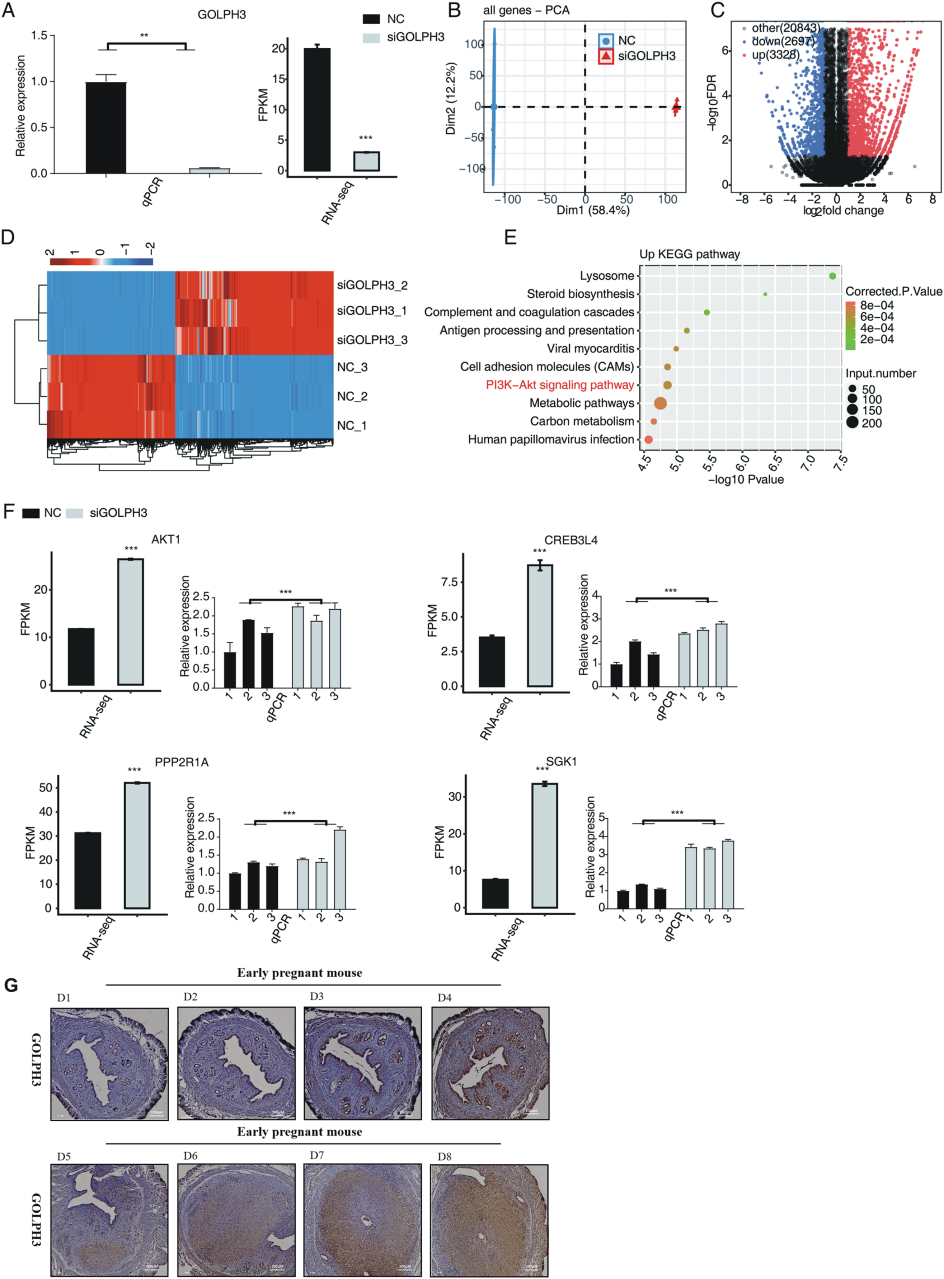
The GOLPH3 regulated transcriptome in hESCs. (A) The histogram showed the RT-qPCR (lift) and the expression pattern and statistical difference of DEGs for GOLPH3 (right) results of control and treatment samples. Error bars represent mean ± SEM. **P-value < 0.01. ***P-value < 0.001. (B) Principal component analysis (PCA) of two groups of samples based on normalized gene expression level. (C) Volcano plot showing all differentially expressed genes (DEGs) between treatment and control samples with DEseq2. The *P* value for correction <0.05 and FC (fold change) ≥2 or ≤0.5. (D) Hierarchical clustering heat map showing expression levels of all DEGs. (E) The top 10 representative KEGG pathway of up-regulated genes. (F) Bar plot showing the expression pattern and statistical difference of DEGs for some important genes. Error bars represent mean ± SEM. ***P-value < 0.001. (G) Golph3 expression was detected in D1–D8 tissue slices of early mouse embryos.

From the immunohistochemical results, it was shown that the expression of GOLPH3 protein in uterine tissues was relatively low and there was no significant difference in the 1–4th days of pregnancy. On the fifth day of pregnancy in mice, GOLPH3 protein was expressed in the epithelium, as the decidua tissue gradually formed. The expression of GOLPH3 protein could be clearly observed on the 6–8th days of pregnancy, and the expression of GOLPH3 gradually increased with the increase of the number of gestational days, and it was mainly expressed in decidua tissue (Fig. 1G). In order to further study the changes of gene expression pattern and level caused by GOLPH3, we transfected the small interfering RNA (siRNA) targeting GOLPH3 into hESCs, in order to knock down GOLPH3 protein expression levels in hESCs cells. As shown in the left panel of Fig. 1A, compared with the control group, RT-qPCR experiment showed that *GOLPH3* mRNA expression level in the knockdown group (siGOLPH3) was significantly lower (Fig. 1A). In order to study the globally transcriptional regulation mediated by GOLPH3, we prepared cDNA libraries (each containing three biological replicates) using RNA samples from siGOLPH3 group and control group, performed RNA-seq experiment. Each sample was sequenced to produce a reading length of 150 nucleotides at both ends. After aligning quality filtered reads to human genome using HISAT2 (Kim, Langmead & Salzberg, 2015) software, we calculated the expression levels of detected genes using FPKM value. From the RNA-seq result, the expression of GOLPH3 was also significantly decreased in siGOLPH3 group (Fig. 1A, right panel). Through principal component analysis (PCA) of all detected genes from siGOLPH3 and control groups, we found that the three samples of the siGOLPH3 group were gathered together, and the three samples of the control group were gathered together; the siGOLPH3 group and the control group could be obviously separated in the first principal component (Fig. 1B), indicating that siGOLPH3 experiment brought significant changes to the overall gene expression pattern. It is suggested that GOLPH3 can significantly change the overall expression characteristics of hESCs transcriptome, which is a potential function of GOLPH3.

### GOLPH3 significantly affects the expression levels of thousands of genes in hESCs

On the basis of obtaining high-quality RNA-seq data, we used DESeq2 software (Love, Huber & Anders, 2014) to obtain differentially expressed genes (DEGs) regulated by GOLPH3 between siGOLPH3 group and negative control group. From the volcanic plot (Fig. 1C) after knocking down GOLPH3 in hESCs cells, DEGS analysis found significant changes in the expression levels of a large number of genes compared with wild-type hESCs cells, including 2,697 downregulated genes and 3,328 upregulated genes. It should be noted that the expression levels of all DEGs between siGOLPH3 group and control group were consistent, which could be found from the clustering heatmap of all DEGs (Fig. 1D), indicating the reliability and consistency of DEGs by siGOLPH3 treatment. We then performed KEGG analysis on the up-regulated genes after siGOLPH3. Enrichment analysis obtained 68 related pathways with corrected *P*-value less than 0.05. The top 10 enriched pathways were lysosome, steroid biosynthesis, complement and coagulation cascade, cell adhesion molecules (CAMs), antigen processing and presentation, viral myocarditis, PI3K-Akt signaling pathway, metabolic pathway, carbon metabolism, and human papillomavirus infection (Fig. 1E). Among these enriched pathways, we found 74 up-regulated genes were from PI3K-Akt signaling pathway. By comparing the expression levels and consistency of these 74 genes in different samples, we found *AKT1*, *CREB3L4*, *PPP2R1A*, and *SGK1* play important roles in the process of endometrial decidualization. To further confirm their altered expression levels by siGOLPH3, we performed RT-qPCR experiment and found the high consistency with the RNA-seq data (Fig. 1F).

### GOLPH3 regulates the expression of transcription factors in hESCs

**Figure 2:**
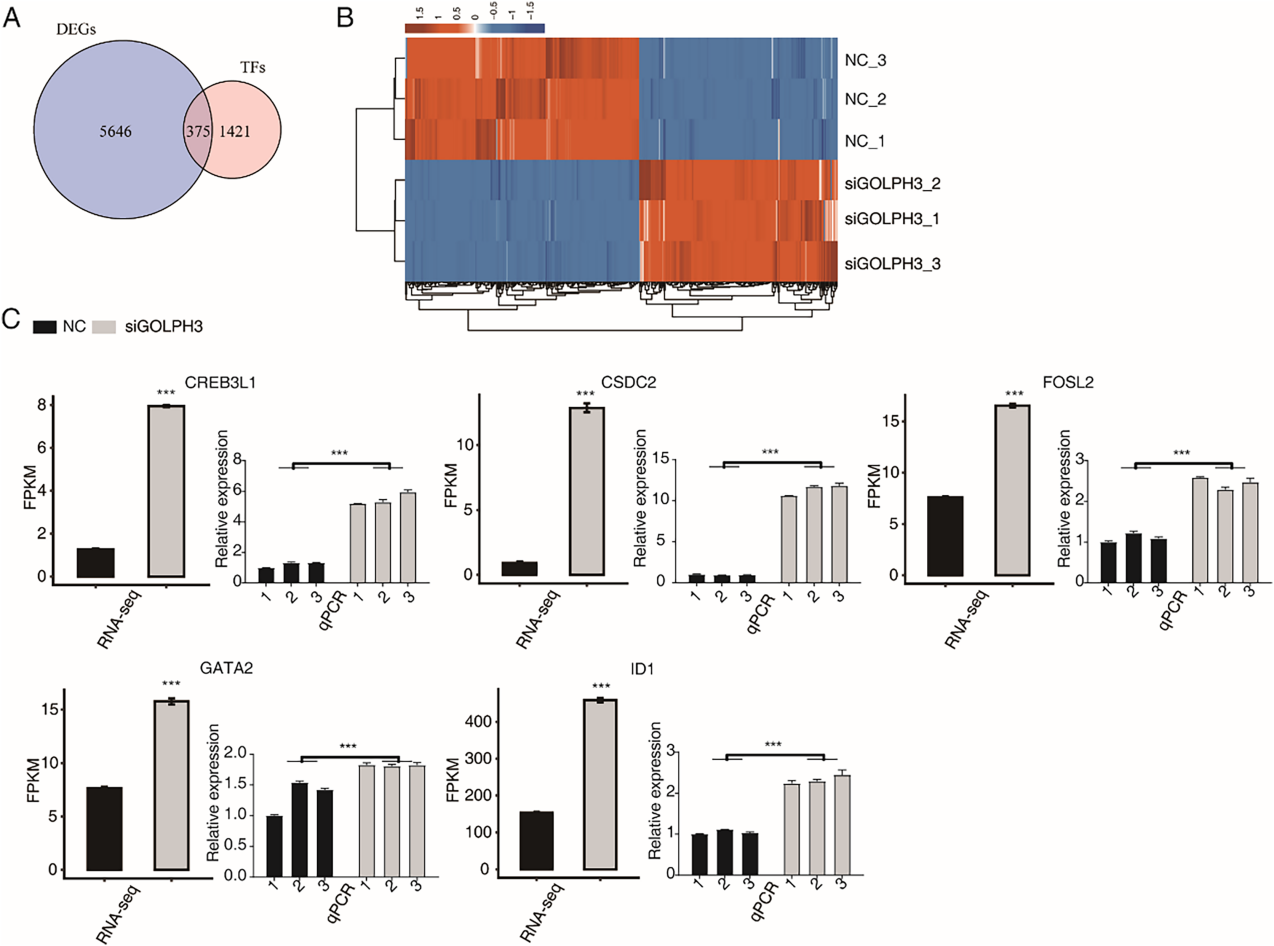
The GOLPH3 regulates the differential expression of TFs in HESC cells in the transcriptome. (A) Venn diagram showing the overlapped genes between TFs and DEGs. (B) Hierarchical clustering heat map showing expression levels of all DE TFs. (C) Bar plot showing the expression pattern and statistical difference of DE TFs. Error bars represent mean ± SEM. ***P-value < 0.001.

KEGG analysis was performed on the down-regulated genes after GOLPH3 silencing, and the genes were enriched into the biological processes such as ribosomal genesis, RNA transport, transcription factors, spliceosomes, Fanconi anemia pathway, RNA degradation, cell cycle, amino acid biosynthesis, endoplasmic reticulum protein processing, selenium compound metabolism. We are concerned that GOLPH3 affected the expression of a large number of transcription factors that were enriched in related pathways. Previous studies have reported that GOLPH3 can act as a scaffold protein to regulate and activate the JAK2-STAT3 pathway, thereby regulating the effect on cell proliferation (Wu et al., 2018), indicating that GOLPH3 can interact with transcription factors (TFs). Therefore, we focused our attention to TFs with differential expression levels in this project. We overlapped 6,025 DEGs in this study with 1,796 known human TFs (Lambert et al., 2018; Hu et al., 2019), and found that GOLPH3 significantly regulated the expression of 375 TFs (Fig. 2A), of which 172 TFs were up-regulated and 203 TFs were down-regulated. We showed the expression of 375 differentially expressed TFs in different samples through heat map. It was found that the down-regulated and up-regulated TFs were similar in color and the expression level was consistent in the siGOLPH3 group and the control group (Fig. 2B). We tend to pay attention to the TFs with the consistent expression level and the average FPKM was greater than 5 in the siGOLPH3 samples. Through literature research, it is found that the five genes in play very important roles in the process of endometrial decidualization, including ID1 (Deepak et al., 2020), GATA2 (Mazur et al., 2015), CREB3L1 (Ahn et al., 2016), CSDC2 (Mazur et al., 2015; Vallejo et al., 2018), and FOSL2 (Mazur et al., 2015) (Fig. 2C). We also performed RT-qPCR experiment for these four five TFs to validate their changed expression levels in siGOLPH3 samples (Fig. 2C). We speculate that GOLPH3 may regulate gene expression by interacting with TFs. Therefore, it plays an important regulatory role in the occurrence of endometrial deciduation.

### GOLPH3 selectively modulates alternative splicing of genes in hESCs

**Figure 3:**
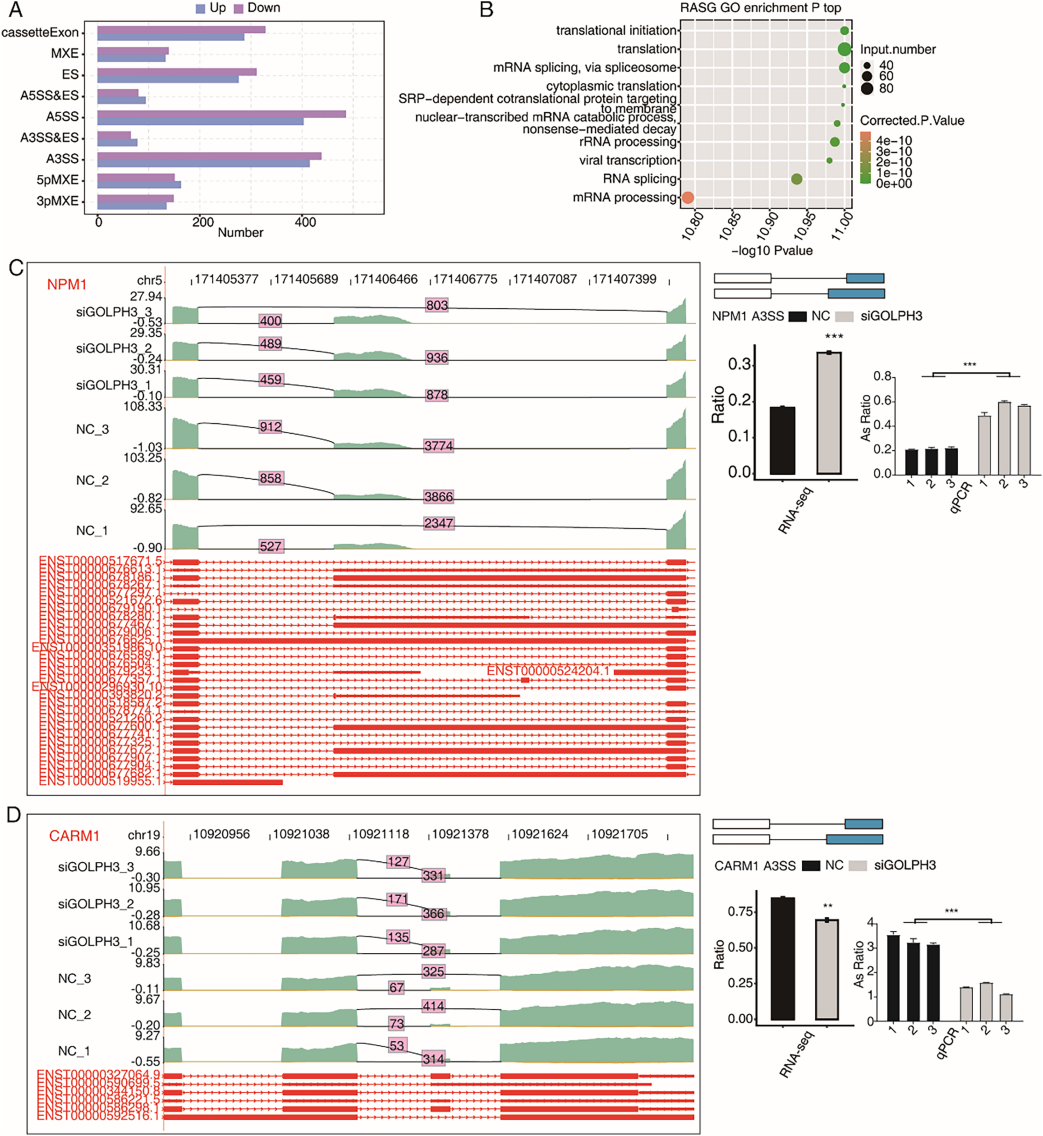
GOLPH3 selectively regulates the alternative splicing of genes in HESCs. (A) Bar plot showing the GOLPH3 regulated alternative splicing event (RASE). X-axis: RASE number. Y-axis: the different types of AS events. (B) Bubble Diagram exhibiting the most enriched GO biological process results of the regulated alternative splicing genes (RASGs). (C) GOLPH3 regulates alternative splicing of NPM1. IGV-sashimi plots show AS changes in GOLPH3 knockdown cells and control cells (left), and the transcripts for the gene are shown below. The schematic diagrams depict the structures of ASEs (right, top). The constitutive exon sequences are denoted with white boxes, intron sequences with horizontal line, while alternative exons with blue boxes. RNA-seq quantification and RT-qPCR validation of ASEs are shown at the bottom of the right panel. The altered ratio of AS events in RNA-seq was calculated using the formula: alternative splice junction reads/(alternative splice junction reads + model splice junction reads). Error bars represent mean ± SEM. ***P-value < 0.001, **P-value < 0.01. (D) GOLPH3 regulates alternative splicing of CARM1.

This study not only discussed the effect of GOLPH3 on gene expression level, but also analyzed the alternative splicing events in hESCs. In the RNA-seq data, 38.25–41.48% were fragments across splice sites. In order to more comprehensively study the changes of alternative splicing events (ASEs), we used ABLas software to analyze ASEs in RNA-seq data. Of the six samples involved in this study, we detected 40,849 known ASEs and 52,390 novel ASEs. In order to analyze GOLPH3-regulated alternative splicing events (RASEs) in the project, we use *t*-test method to evaluate whether there is a significant difference of ASEs by setting the criteria as *P*-value < 0.05. In this study, 4,131 non intron retention (NIR) ASEs regulated by GOLPH3 were detected, including 889 A5SS, 853 A3SS, 615 cassette exon, and 587 ES. In addition, there were five other events with a smaller number, including 284 3PMXEs, 314 5PMXEs, 143 A3SS&ESs, 174 A5SS&ESs, and 272 MXEs (Fig. 3A). Moreover, we found that the genes with RASEs (RASGs) were enriched in the pathways related to RNA metabolism and protein translation, such as translation initiation, translation, mRNA splicing *via* spliceosomes, cytoplasmic translation, mRNA catabolic processes of nuclear transcription, SRP-dependent co-translational proteins targeting membranes, rRNA processing, RNA splicing, and mRNA processing (Fig. 3B). According to the significance of the ratio value of RASEs between samples and their correlation with endometrial decidualization, we found that NPM1 is a nucleolar stress factor (Liang et al., 2020), ADAM15 participates in uterine implantation remodeling (Kim et al., 2006), GNAS participates in endometrial function (Meng et al., 2014), and CARM1 participate in decidualization (Zhang et al., 2021). We could observe that the proportion of RASEs of these four genes were significantly changed in the siGOLPH3 group, and were consistently validated by RT-qPCR experiment (Figs. 3C, 3D, S1A and S1B).

### GOLPH3 selectively regulates alternative splicing of TFs in hESCs

**Figure 4:**
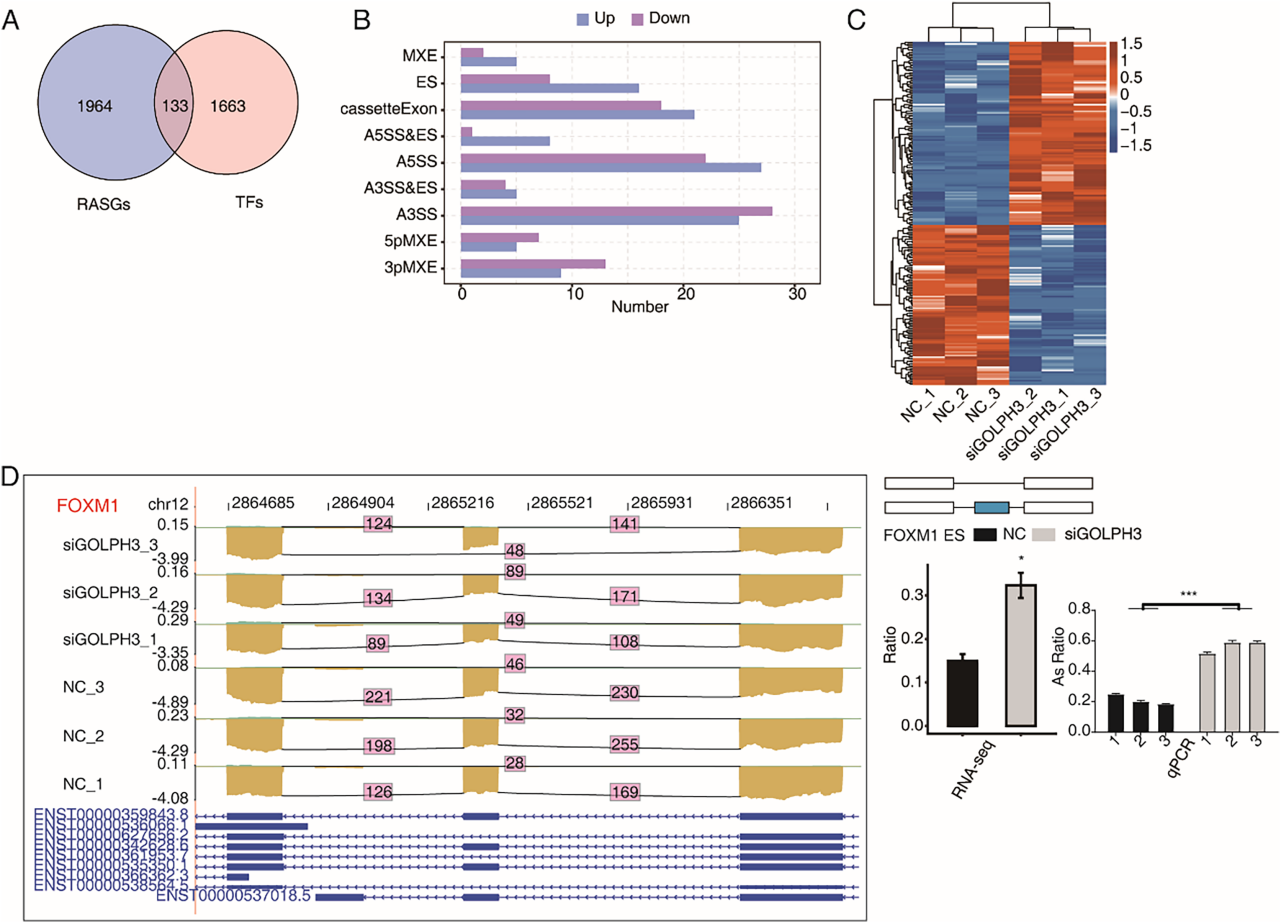
GOLPH3 selectively regulates the alternative splicing of TFs in HESC cells. (A) Venn diagram showing the overlapped genes between RASGs and TFs. (B) Bar plot showing the GOLPH3 regulated alternative splicing event (RASE) of TFs. (C) Hierarchical clustering heat map showing the ratio values of the DE TFs RASE. (D) GOLPH3 regulates alternative splicing of FOXM1. IGV-sashimi plots show AS changes in GOLPH3 knockdown cells and control cells (left), and the transcripts for the gene are shown below. The schematic diagrams depict the structures of ASEs (right, top). The constitutive exon sequences are denoted with white boxes, intron sequences with horizontal line, while alternative exons with blue boxes. RNA-seq quantification and RT-qPCR validation of ASEs are shown at the bottom of the right panel. The altered ratio of AS events in RNA-seq was calculated using the formula: alternative splice junction reads/(alternative splice junction reads + model splice junction reads). Error bars represent mean ± SEM. ***P-value < 0.001, *P-value < 0.05.

A large number of TFs were observed to be differentially expressed in the above part. We also pay attention to the TFs with RASEs in this study. When an ASE of TF was changed significantly, it may further affect the expression level of other genes. We overlapped 2,097 RASGs with 1,796 TFs and found that 224 RASEs from 133 TFs were changed significantly (Fig. 4A), including 121 up-regulated RASEs and 103 down-regulated RASEs (Fig. 4B).The heat map was used to show the changes of 224 RASEs in the six samples. It could be seen that the consistency of RASEs were also consistent among the three replicates (Fig. 4C). Figure 4D showed the visual distribution of alternative spliced reads of FOXM1 (Liang et al., 2020), which was significantly increased of exon skipping after siGOLPH3, and was validated by RT-qPCR experiment (Fig. 4D). The RASE of FOXM1, and its effects on the expression of downstream genes may play an important role in the process of decidualization (Xie, Cui & Kong, 2014).

## Discussion

As part of normal pregnancy and maintaining the fetal-maternal interface, endometrium decidualization plays an important role (Gellersen, Brosens & Brosens, 2007). We previously established an *in vitro* endometrial decidualization mouse model. GOLPH3 expression levels in mouse uterine tissues also increased with gestation days, suggesting that GOLPH3 may have potential regulatory functions. As a protein exporter from the endoplasmic reticulum, GOLPH3 is important for modifying and classifying proteins (Dippold et al., 2009). In the study of endometrial devascularization and related fields, it was found that the copy number variation of GOLPH3 increased the risk of recurrent abortion (Nagirnaja et al., 2014). Furthermore, GOLPH3 expression in ovarian granulosa cells can enhance the selection of high-quality embryos and increase pregnancy rates (Lin et al., 2017). However, it is unknown how GOLPH3 contributes to the devascularization of the endometrium and what its underlying mechanisms are. Therefore, we hypothesize that GOLPH3 may be involved in modulating alternative splicing levels and gene expression levels during endometrial decidualization by interacting with other factors.

### GOLPH3 regulates the expression levels of a large number of genes in hESCs

The present study explored the transcriptional data of GOLPH3, and discovered the potential regulatory functions at transcriptional and post-transcriptional levels. Specifically, the silencing operation of GOLPH3 (siGOLPH3) in hESCs significantly changed the expression of 6,025 genes and 4,131 alternative splicing events. Regulation of gene expression occurs primarily at the transcriptional level, while the alternative splicing is mainly regulated at the post-transcriptional level. The results of siGOLPH3 on these two levels showed that GOLPH3 has new functions and regulatory modes in addition to its inherent protein modification and transportation functions.

A number of important pathways were enriched in the DEGs regulated by GOLPH3. PI3K/Akt signaling pathway is a key pathway worthy of attention, including AKT1/AKT, CREB3L4/CREB3, PPP2R1A, and SGK1. It has been reported that PI3K/Akt signaling is involved in endometrial decidualization (Salker et al., 2016). AKT is an emerging intracellular mediator in hESCs. Through cAMP/PKA signal transduction pathway, AKT phosphorylation may regulate the function of hESCs with decidualization. In addition, treatment of hESCs with AKT activator SC-7 can restore the reduced migration and decidualization of hESCs under simulated microgravity (SM) (Cho et al., 2019). CREB3/Luman is a member of CREB3 subfamily. Knockdown of Luman leads to a large number of gene expression disorders during decidualization of ESCs (Zhao et al., 2020). Hormone treatment increased the expression of CREB3 regulatory factor (CREBRF), and knockdown of CREBRF hindered the proliferation of ESCs by arresting the cell cycle in S-phase, thus affected endometrial decidualization (Yang et al., 2018).

There are few literatures about another important gene PPP2R1A in decidualization of endometrium, but the conclusion reported in one study is of great significance for the study of endometrium’s molecular mechanism: WNK1 is very important for decidualization of hESCs *in vitro*. WNK1 interacts with PPP2R1A to maintain the level of PP2A subunit and stabilize its activity, and then dephosphorylates AKT (Chi et al., 2020). SGK1, is a kinase participated in epithelial ion transport and cell survival, is up-regulated in unexplained infertility, most notably in the intraluminal epithelium, but down-regulated in the endometrium of women with recurrent abortion RPL. Relative SGK1 deficiency also indicates decidualized RPL stromal cells in human subjects and makes them more susceptible to oxidative stress (Salker et al., 2011). A down-regulated SGK1 in the endometrium promotes embryo implantation, whereas a constitutively active mutation in the mouse uterus inhibits implantation. The balanced activities of two related serine/threonine kinases AKT and SGK1 critically control the implantation process (Salker et al., 2016). The above existing conclusions show that GOLPH3 has an important regulatory function on PI3K/Akt pathway, which may be a mode of action of GOLPH3 on endometrial decidualization.

### GOLPH3 selectively regulates alternative splicing of transcription factors in hESCs

The results of this study demonstrated differential expression of numerous transcription factors. We also pay attention to the TFs with alternative splicing events (ASEs) in this study. The ASEs of TFs may further affect the expression level of other genes. We analyzed the overlap between 2,097 RASGs and 1,796 TFs, and found that 224 RASEs of 133 TFs were significantly changed. Therefore, we also looked at the role of GOLPH3 in alternative splicing regulation. It was found that NPM1 is a nucleolar stress factor, ADAM15 is involved in uterine implantation remodeling, GNAS is involved in endometrial function, and CARM1 is involved in decidualization. These four genes involved in endometrial function have significant RASEs with high ranking. At the same time, it has been reported that implantation of embryos is related to nucleolar stress (Liang et al., 2020). ADAM family proteins are mainly located in the lumen or glandular epithelial layer, and their intensity varies according to the type of Adam and the progress of pregnancy. In particular, ADAM-8, -12 and -15 are mainly located at the implantation site of uterine tissue, suggesting that mouse ADAMs may contribute to uterine remodeling during implantation (Kim et al., 2006). The function of GNAS can be inhibited by GnRH antagonist, which is very important for endometrial function (Meng et al., 2014). Alternative splicing regulation is a very important post-transcriptional regulation in eukaryotes, which contributes significantly to functional diversity of proteins (Modrek & Lee, 2002). The regulation of alternative splicing by GOLPH3 can change the translation efficiency, protein level and protein function of regulated genes. We speculate that the protein function of alternative splicing genes NPM1 and ADAM15 regulated by GOLPH3 has changed after GOLPH3 silencing, which has an impact on endometrial de membrane. Therefore, this regulatory function may be another mode of action of GOLPH3 on endometrial demineralization, and the specific biological function needs to be further explored.

As a Golgi phosphorylated protein, GOLPH3 does not have the classical transcriptional regulation function; It can regulate the expression of 6,025 genes, suggesting that this transcriptional regulation function may be executed by an indirect role through other transcriptional regulators. Therefore, we focused on the TFs in DEGs and RASGs, and found 375 differentially expressed TFs and 133 differentially spliced TFs. Through the analysis of these differential TFs, we locked in seven transcription factors associated with endometrial demineralization, including ID1, GATA2, CREB3L1, CSDC2, FOSL2, FOXM1 and TCF3. A lack of ID1 expression may impair decidualization of stromal cells (Deepak et al., 2020). FOSL2 may be an important transcriptional co-regulatory factor of PGR by directly interacting with the gene regulatory region positively regulated in the process of decidualization (Mazur et al., 2015). During implantation, GATA2 protein is expressed within the uterus and glandular epithelium, and has spatiotemporal homology with progesterone receptor (PR). In addition, The expression of GATA2 in decidualized matrix is continuous throughout early pregnancy, indicating that GATA2 is involved in the maintenance of decidual cells (Rubel et al., 2012). ChIP-seq and RNA-seq experiments in hESCs before and after ecdysis *in vitro* showed that GATA2 could bind to PGR and affect the expression of a large number of genes, regulate decidualization of endometrial stromal cells, especially through WNT activation and stem cell differentiation (Kohlmeier et al., 2020). Intrauterine siRNA injection can inhibit the abnormal expression of CSDC2 in early pregnancy; compared with control siRNA injected animals, atrial area expansion and M dysplasia were produced (Vallejo et al., 2018). Both mice and humans require CREB3L1 for decidualization, the gene that targets the progesterone receptor (PR) (Ahn et al., 2016). Human endometrium expresses FOXM1 dynamically during menstruation. When FOXM1 expression was reduced, hESCs could not differentiate into estrogen, progesterone, or dbcAMP (Liang et al., 2020). A conditional deletion of FOXM1 in mice causes implantation failure and regional decidual defects, such as smaller membrane oysters with increased SDZ (Xie, Cui & Kong, 2014). At the same time, FOXM1 can activate the transcription of STAT3, so as to ensure normal hESCs differentiation (Liang et al., 2020). In addition, studies have also confirmed that TCF3 regulates endometrial decidualization and uterine hemoglobin biosynthesis in humans and mice, suggesting that the uterus, as a non-erythroid hemoglobin biosynthetic organ, can respond to the transcriptional activity of TCF3. Based on the above discussion, we speculate that GOLPH3 may regulate gene expression by changing transcription factors and alternative splicing, and then play a role in endometrial decidualization.

The process of embryo implantation is characterized by the proliferation and differentiation of endometrial cells and vascular remodeling to support the growing embryo, which is very similar to the invasion and metastasis of tumor cells. According to studies, GOLPH3 is important for proliferation, differentiation, and metastasis of tumor cells in various types of tumors (Ognibene et al., 2019; Wang et al., 2015; Sun et al., 2017). This is closely related to the physiological function of GOLPH3. GOLPH3 can maintain Golgi homeostasis and promote vesicle formation and transport. It also regulates the overall mitochondrial mass and affects cell metabolism by delivering cardiolipin and mitochondrial lipids. Coupled to actomyosin dynamics *via* phosphatidylinositol signaling, the protein plays a key role in cell division and migration. At the same time, GOLPH3 also participate in tumor angiogenesis and promote its rapid growth (Li et al., 2015; Peng et al., 2014). During organ development, GOLPH3 directly regulates Tctp-Rheb-mTORC1 axis activity, thereby affecting cell growth and cell number (Frappaolo et al., 2022). Drosophila spermatocytes dividing during cytokinesis are associated with membrane trafficking coupled to contractile ring assembly *via* PI(4)P-GOLPH3 (Sechi et al., 2021). In the process of embryo implantation, the semi-allogeneic embryo needs to locate, adhere to and invade the uterine tissue, showing biological behaviors similar to tumor cells, and affecting cell proliferation, division, apoptosis, and angiogenesis in the uterus (Dai et al., 2015; Liu et al., 2018). Physiological functions of GOLPH3 may be involved in embryo implantation. For the future, we will further investigate the molecular regulation mechanism of GOLPH3 in decidualization.

## Conclusion

By silencing GOLPH3 in human endometrial stromal cells, the transcriptome data affected by GOLPH3 were obtained by high-throughput sequencing technology. Differentially expressed genes and genes involved in differential alternative splicing events were found to be enriched in the PI3K/Akt pathway. Through this part of research, we expanded our understanding of the function of GOLPH3, further promoted and clarified its mode of action in the occurrence and development of endometrial decidualization, and pointed out the direction for the further study of the mechanism and influence pathway of GOLPH3 on endometrial decidualization.

## Supplemental Information

10.7717/peerj.15048/supp-1 Supplemental Information 1 RT-PCR raw data for Figure 1A.

10.7717/peerj.15048/supp-2 Supplemental Information 2 GOLPH3 selectively regulates the alternative splicing of genes in hESCs. GOLPH3 regulates alternative splicing of GNAS. IGV-sashimi plots show AS changes in GOLPH3 knockdown cells and control cells (left), and the transcripts for the gene are shown below. The schematic diagrams depict the structures of ASEs (right, top). The constitutive exon sequences are denoted with white boxes, intron sequences with horizontal line, while alternative exons with blue boxes. RNA-seq quantification and RT-qPCR validation of ASEs are shown at the bottom of the right panel. The altered ratio of AS events in RNA-seq was calculated using the formula: alternative splice junction reads/(alternative splice junction reads + model splice junction reads). Error bars represent mean ± SEM. ***P-value < 0.001, **P-value < 0.01, *P-value < 0.05.

## Additional Information and Declarations

### DNA Deposition

The following information was supplied regarding the deposition of DNA sequences: The data is available at NCBI: GSE215155. https://www.ncbi.nlm.nih.gov/geo/query/acc.cgi?acc=GSE215155.

### Data Availability

The following information was supplied regarding data availability: The data is available at NCBI: GSE215155, PRJNA889007. https://www.ncbi.nlm.nih.gov/bioproject/PRJNA889007.
