# Supplementary figures and images for: GOLPH3 modulates expression and alternative splicing of transcription factors associated with endometrial decidualization in human endometrial stromal cells
Source: PeerJ. 2023 Mar 20;11:e15048. doi: 10.7717/peerj.15048 (PMC10035422; doi:10.7717/peerj.15048)

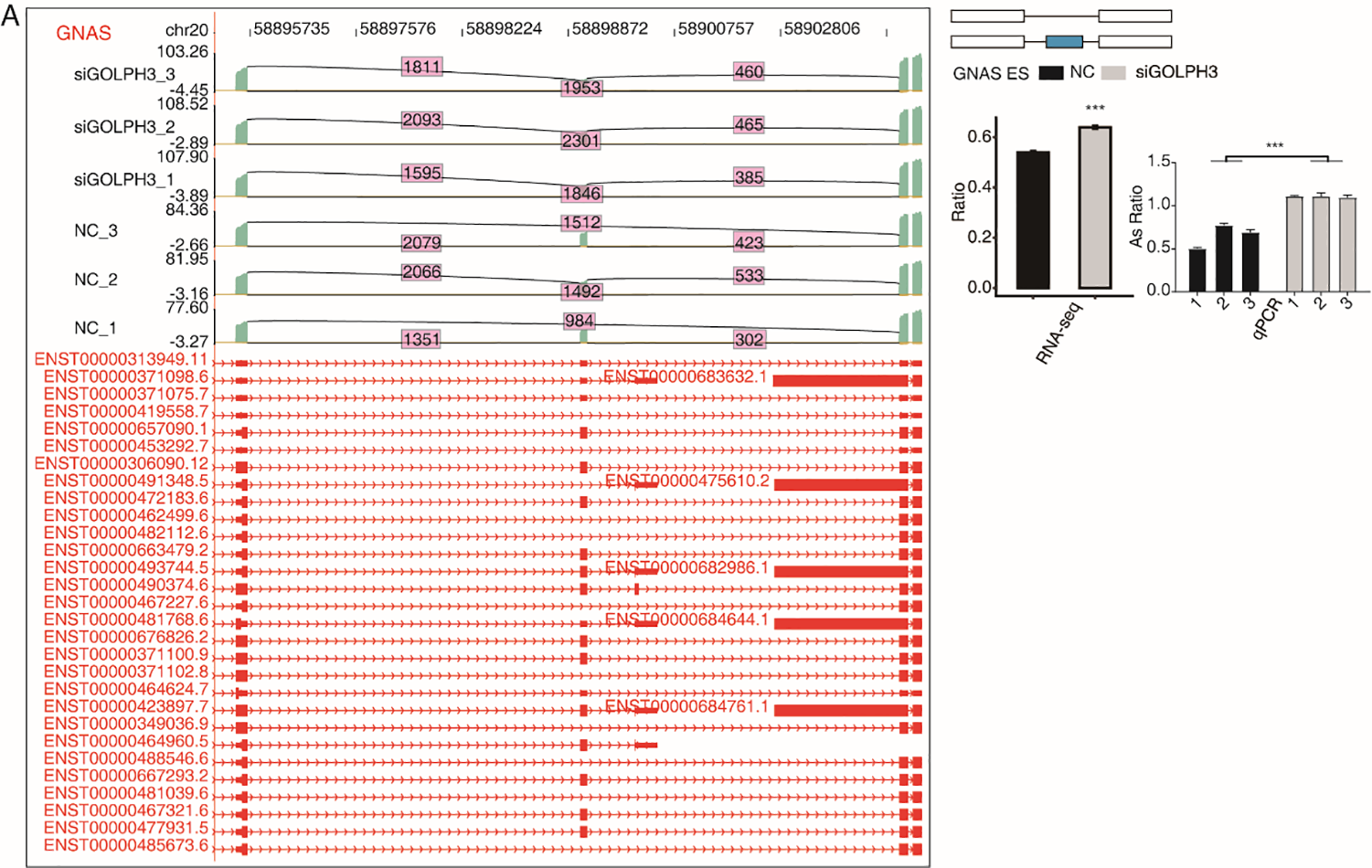

Supplement: Supplemental Information 2 — GOLPH3 regulates alternative splicing of GNAS. IGV-sashimi plots show AS changes in GOLPH3 knockdown cells and control cells (left), and the transcripts for the gene are shown below. The schematic diagrams depict the structures of ASEs (right, top). The constitutive exon sequences are denoted with white boxes, intron sequences with horizontal line, while alternative exons with blue boxes. RNA-seq quantification and RT-qPCR validation of ASEs are shown at the bottom of the right panel. The altered ratio of AS events in RNA-seq was calculated using the formula: alternative splice junction reads/(alternative splice junction reads + model splice junction reads). Error bars represent mean ± SEM. ***P-value < 0.001, **P-value < 0.01, *P-value < 0.05. [file peerj-11-15048-s002.png]
